# Prediction of CpG-island function: CpG clustering vs. sliding-window methods

**DOI:** 10.1186/1471-2164-11-327

**Published:** 2010-05-26

**Authors:** Michael Hackenberg, Guillermo Barturen, Pedro Carpena, Pedro L Luque-Escamilla, Christopher Previti, José L Oliver

**Affiliations:** 1 Dpto. de Genética, Facultad de Ciencias, Universidad de Granada, Campus de Fuentenueva s/n, 18071, Granada, Spain; 2 Lab. de Bioinformática, Centro de Investigación Biomédica, PTS, Avda. del Conocimiento s/n, 18100, Granada, Spain; 3 Dpto. de Física Aplicada II, E.T.S.I. de Telecomunicación, Universidad de Málaga 29071-Malaga, Spain; 4 Division of Sleep Medicine, Brigham and Woman's Hospital, Harvard Medical School, Boston, MA 02115, USA; 5 Dpto. de Ingeniería Mecánica y Minera, EPS Jaén-Universidad de Jaén, Campus Las Lagunillas s/n A3-008, 23071-Jaén, Spain; 6 Computational Biology Unit, Bergen Center for Computational Science & Sars Centre for Marine Molecular Biology, University of Bergen, Thormøhlensgt 55, 5008 Bergen, Norway

## Abstract

**Background:**

Unmethylated stretches of CpG dinucleotides (CpG islands) are an outstanding property of mammal genomes. Conventionally, these regions are detected by sliding window approaches using %G + C, CpG observed/expected ratio and length thresholds as main parameters. Recently, clustering methods directly detect clusters of CpG dinucleotides as a statistical property of the genome sequence.

**Results:**

We compare sliding-window to clustering (i.e. *CpGcluster*) predictions by applying new ways to detect putative functionality of CpG islands. Analyzing the co-localization with several genomic regions as a function of window size *vs*. statistical significance (*p-value*), *CpGcluster *shows a higher overlap with promoter regions and highly conserved elements, at the same time showing less overlap with *Alu *retrotransposons. The major difference in the prediction was found for short islands (CpG islets), often exclusively predicted by *CpGcluster*. Many of these islets seem to be functional, as they are unmethylated, highly conserved and/or located within the promoter region. Finally, we show that window-based islands can spuriously overlap several, differentially regulated promoters as well as different methylation domains, which might indicate a wrong merge of several CpG islands into a single, very long island. The shorter *CpGcluster *islands seem to be much more specific when concerning the overlap with alternative transcription start sites or the detection of homogenous methylation domains.

**Conclusions:**

The main difference between sliding-window approaches and clustering methods is the length of the predicted islands. Short islands, often differentially methylated, are almost exclusively predicted by *CpGcluster*. This suggests that *CpGcluster *may be the algorithm of choice to explore the function of these short, but putatively functional CpG islands.

## Background

The methylation of CpG dinucleotides is an important epigenetic modification of DNA, required in mammals for embryonic development, genomic imprinting and X-chromosome inactivation [1-3]. Around 80% of all CpG dinucleotides are methylated in mammal genomes. The exceptions are short stretches of CpG dinucleotides (CpG islands or CGIs), which are predominantly hypomethylated in healthy tissues [4,5]. CGIs are thought to be predominantly located in the promoter region of genes; around 70% of all genes have a CGI overlapping its promoter region. Moreover, virtually all housekeeping genes are associated to CGIs, while only half of the tissue specific genes show such association [6]. Given its location in the promoters, CGIs may play important roles in the regulation of gene expression. An example is the aberrant methylation of CGIs observed in many cancer types [7-11]. Moreover, evidence exist that the differential or tissue specific methylation of CpG islands may be involved in the regulation of tissue specific genes [12].

Accurate prediction tools are therefore needed and a considerable effort has been carried out over the last decade to detect CGIs in mammal genomes. Many different algorithms have been proposed, most of them based on the criteria of Gardiner-Frommer [1]. These authors proposed in 1987 thresholds for the detection of CGIs: GC-content (50%), CpG observed/expected (O/E) ratio (0.6) and length (200 bp). Many of the published methods simply readjust these thresholds. However, it has been shown that filtering criteria-based definitions of CpG islands are mathematically incomplete and non-operational, as the sliding window methods frequently fail to identify a large percentage of subsequences that meet the filtering criteria [13].

Recently, methods based on the clustering of CpGs along the genome sequence detect CGIs as a statistical property, thereby not relying on thresholds of GC-content, O/E ratio and length. The first algorithm published in this category was the *CpGcluster *method [14], which detects the CGIs by means of the distances between CpGs, then assigning a statistical significance to each cluster of CpG dinucleotides. Subsequently, *CpGcluster *was followed by other methods detecting CGIs by means of the CpG densities [15-18]. In the same way, many other features could also contribute to determine the boundary of individual CpG-islands, such as transcription factors and nucleosome location. The nucleosome code could be an important ingredient of future CGI models, although sequence features will probably remain as the principal component (see, for example, [19]). Epigenetic information may be also of help in detecting CGIs by making use of contextual information [20].

Given the conceptual differences between sliding window algorithms (SWA) using a high parameter space and those detecting CGIs as a statistical property of the CpG clustering in DNA sequences, disagreement exist on the way CGIs should be predicted. Recently, a comparison between islands detected by the window-based Takai-Jones (TJ) program [21] and those detected by *CpGcluster *was published [22]. The comparison evaluated mainly the co-localization of CGIs and known promoters and concludes an overall advantage for the TJ approach over *CpGcluster*.

We present here new ways to detect putative function of CGIs, emphasizing the basic difference between *CpGcluster *and SWA predictions: the statistical significance introduced by *CpGcluster *instead of the conventional length threshold. We show that the statistical significance assigned to each *CpGcluster *island is a key criterion to control the overlap with promoter regions, evolutionarily conserved elements and spurious *Alu *elements. Finally, we show that many short (<200 bp) islands (CpG islets) may be also functional, given its overlap with either promoter or evolutionary conserved regions and the absence of methylation in at least one tissue. As many of these islets are exclusively predicted by *CpGcluster*, this may be the algorithm of choice for experimental essays aimed to verify the function of these short islands.

## Results and Discussion

**Figure 1:**
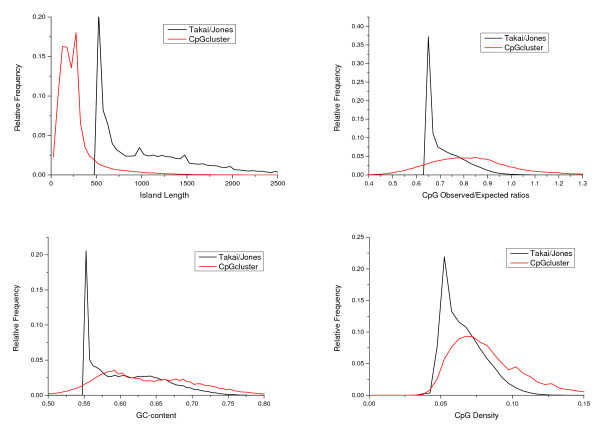
**Comparison of the distributions of the island length for both the *CpGcluster *and Takai/Jones algorithm (top left); the observed to expected ratios of CpG frequencies (top right); the island GC-content (bottom left); and the island CpG density (bottom right)**. It can be seen that, for all this four properties, the SWA distributions are heavily biased towards their respective thresholds. However, *CpGcluster *distributions do not show this artifact.

The way sliding-window approaches and *CpGcluster *detect CGIs are conceptually different. While SWA detect regions above the thresholds of G + C, O/E, min CpG and length, *CpGcluster *predicts statistically significant clusters of CpGs as CGIs. As a first consequence, the statistical properties of the predicted islands are different as well (Figure 1); e.g. in SWA approaches the distributions of important CGI properties like %G + C and O/E ratio are heavily biased towards the user thresholds.

Therefore, the first part of this work is basically aimed to clarify: 1) the differences between the length threshold used by SWA and the statistically significance used by *CpGcluster*; and 2) the consequences that the differences in the number of predicted islands and the mean length might have on the prediction quality.

Prediction quality has been assigned conventionally by the percentage of overlap with promoter regions and spurious *Alu *elements. In the original publication of *CpGcluster *[14] we added the overlap with evolutionarily conserved elements or PhastCons [23] as an indicator of putative functionality. Here, we add several new types of analysis to assess the prediction quality, namely the capability to distinguish between different methylation domains or different alternative Transcription Start Sites (TSSs).

### CpG islands in the promoter region

Since CpG islands are preferentially located in the promoter region of genes, this fact has been extensively used to assess the quality of CGI predictions [24]. Recently, it has been claimed [22] that a higher percentage of TJ islands (35%) are located within the promoter when compared to *CpGcluster *islands (14.7%). In Table 1, we show a similar analysis as carried out in [22], but extending the comparison to other window based programs and different prediction sets for the *CpGcluster *algorithm. When considering *CpGcluster *islands with *p-value *≤ 1E-5 (the original relaxed set), the CGI fraction overlapping the promoter region is effectively smaller than for the other programs. However, note that the numbers of CGIs predicted by window-based methods are far below the number predicted by *CpGcluster*. To allow for an unbiased comparison, we obtained a second, strict set of *CpGcluster *islands simply by increasing the required statistical significance to *p-value *≤ 1E-20 (i.e. filtering out the less significant islands), then obtaining a total 25,454 CGIs. This number is within the range of recent estimates for the complete human somatic cell CGI complement [25]. The strict, more statistically significant set of *CpGcluster *islands shows now the highest overlap (52.4%) with the promoter region. This advantage looks even more important when considering that the genome coverage of our strict set (0.65%) was the lowest one. This indicates a high specificity of *CpGcluster*, which strongly supports our original claim that the *p-value *is the most important parameter to distinguish promoter CGIs from the rest of genome islands [14].

**Table 1:** Co-localization of CpG islands and the promoter region.

Method	Number of predicted islands	Genome coverage (%)	Promoter overlap (R13)	
			**Number of islands**	**%**
			
TJ	37,323	1.43	14,034	37.60

UCSC	27,639	0.74	13,369	48.40

CpGproD	76,886	2.81	14,814	19.30

*CpGcluster*:				

relaxed set*	198,702	1.90	30,660	15.43

strict set**	25,454	0.65	13,349	52.40

*p-value ≤ 1E-5;** *p-value *≤ 1E-20

### A comparison of length and *p-value *thresholds

**Figure 2:**
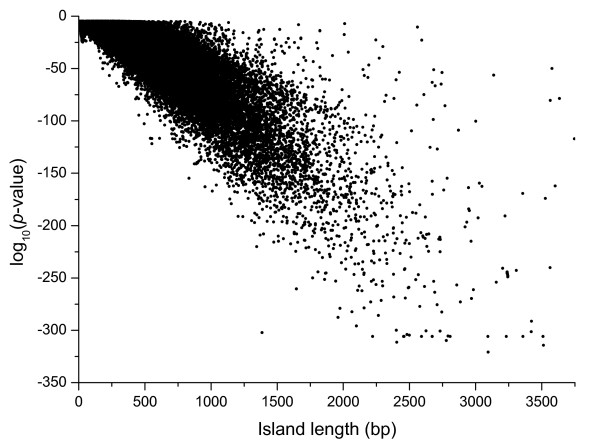
**The length of *CpGcluster *islands vs. the logarithm of the assigned *p-value***. It can be seen that no linear correlation exists and that the relation between *p-value *and length is more complex, e.g. the *p-value *depends on both the island length and the island density.

The main quality parameter in SWA is the window size (CGI length threshold). Originally, the window size was set to 200 bp to assure that the detected regions surpass the G + C and O/E criterion not due to chance alone [1]. Subsequently, this threshold was increased to 500 bp in order to reduce the false positive rate by eliminating spurious *Alu *elements [21]. This criterion was replaced in *CpGcluster *by the statistical significance (*p-value*), a more robust and reliable way to distinguish true CGIs from stochastic noise, disregard island length [14]. Note that the *p-value *is not just a different expression for the island length. A non-linear relation exists between the *p-values *and the lengths of the predicted *CpGcluster *islands, as the *p-value *depends on both the island length and the island density (Figure 2).

**Figure 3:**
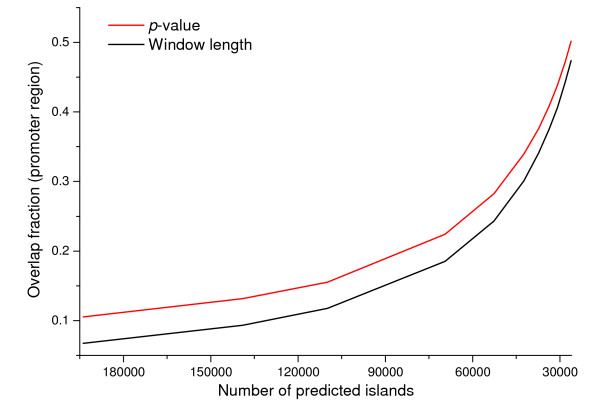
**Variation of the overlap fraction of predicted islands with RefSeq promoter regions**. The different sets of predicted islands have been obtained by varying the *CpGcluster p-value *and the SWA window length.

**Figure 4:**
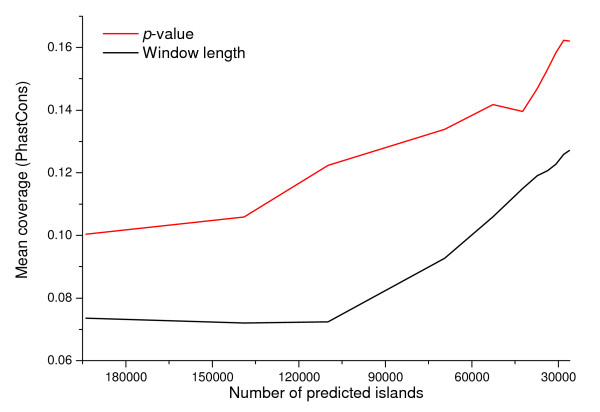
**Variation of the mean coverage by PhastCons in different predicted island-sets obtained by varying the *CpGcluster p-value *and the SWA window length**.

**Figure 5:**
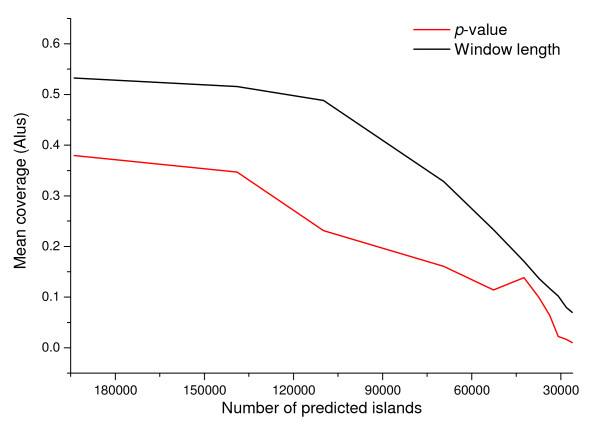
**Variation of the mean coverage by *Alus *in different predicted island-sets obtained by varying the *CpGcluster p-value *and the SWA window length**.

To evaluate the discrimination power of *CpGcluster p-value *against window size, we generated a series of island-set predictions, each one containing the same number of islands, by appropriately varying the window size or the *p-value *thresholds. Next, we determined the overlap of the resulting islands with the promoter regions, PhastCons elements [23] and *Alu *repeats. The island sets selected by *p-value *clearly outperformed those selected by length: a higher percentage of *CpGcluster *islands overlap with promoters (Figure 3) and PhastCons elements (Figure 4) along the entire range of the two parameters, at the same time reducing the overlap with spurious *Alu *elements (Figure 5). Table 2 shows the correspondence between the number of predicted islands, *p-value *and window length.

**Table 2:** Correspondence between the number of predicted islands, log (*p-value*) and window length.

No. of predicted islands	log (*p-value*)	Window length
193,856	5.06509	200

139,013	6.1864	250

109,907	7.19943	300

69,477	9.82744	350

52,687	11.85626	400

42,392	13.73824	450

37,293	14.96788	500

33,691	15.95388	550

30,881	16.8824	600

28,162	18.18919	650

26,192	19.45203	700

The results in Figures 3, 4, 5 are straightforward in comparing the relative strengths of the two main parameters involved in CGI quality (length and *p-value*). The increased stringency in the conventional parameters used by the TJ program excluded contaminating *Alu *elements, but it also reduced the number of gene promoter associated islands, suggesting that bona fide CGIs were also being discarded [25]. However, raising the statistical significance (i.e. decreasing the *p-value*) of *CpGcluster *leads to an exponential increase in the overlap with promoters or PhastCons, simultaneously decreasing the overlap with *Alu *elements. *CpGcluster *algorithm is, therefore, a more rational and powerful way to increase CGI prediction quality. An additional advantage is that *CpGcluster p-value *would be particularly useful in comparative genomics of CGIs, making possible the comparison of CGIs with the same statistical significance, but obtained from different species, despite variations in G + C content or CpG density.

### Prediction of unmethylated regions

The most important criterion to assess putative functionality of a CpG island is the absence of methylation. Therefore, the comparison to experimentally verified, unmethylated regions is another important analysis type to establish prediction quality.

Recently, the methylation status of 697 hypermethylated and 6,987 hypomethylated promoter regions in WI38 primary lung fibroblast [26] have been used to compare the prediction quality of TJ and *CpGcluster *algorithms [22]. In this study, the prediction quality was measured in the following way: i) true positives (TP): hypomethylated promoters containing a predicted island, ii) false positives (FP): hypermethylated promoters containing a predicted island, iii) true negatives (TN): hypermethylated promoters not containing a predicted island, and iv) false negatives (FN): hypomethylated promoters not containing a predicted island.

However, in our opinion, there is an important pitfall in such an approach. It is known that the methylation state of a given region can change among different tissues; therefore, assigning a "false positive" label to a predicted island which has been shown to be methylated in a single tissue may be misleading, as the same prediction could be perfectly "true positive" if measured in a different tissue.

Fortunately, Weber *et al*. [26] also determined the methylation states in sperm. Analyzing fibroblast and sperm data together, we observed that 11,260 regions are unmethylated in both tissues but 1,550 are unmethylated in one tissue but methylated in the other one. This means that around 12% of the regions are differentially methylated; therefore, a substantial number of FPs were actually TPs. Given these data, in our opinion, without the knowledge of the methylation state in a vast number of different tissues, the number of "false positive predictions" cannot be assessed in this way.

We therefore based our quality assessment on sensitivity, a measure not dependent on the false positive rate, as well as on the estimation of the lower bound for the positive predictive value (PPV, see Data and Methods), a measure used in the gene prediction field under the name of specificity [27]. We used two different experimentally validated sets of unmethylated regions (see Data and Methods) to assess the quality of the 5 sets of predicted islands. Table 3 depicts the results when taking genome-wide, experimentally verified unmethylated CpG islands as reference (Bird's islands, [28]). The table shows that the *CpGcluster *relaxed set shows the highest sensitivity while the strict set shows the lowest one. When considering the lower boundary of the PPV (i.e. the method is at least as specific as this value), we observed the contrary pattern, the *CpGcluster *strict set now shows the highest PPV, while the relaxed set shows the lowest one. Table 4 seems to confirm this trend when using unmethylated regions which are mainly related to promoters [26]. These results indicate that *CpGcluster *is either the most sensitive or the most specific algorithm, depending on the applied *p-value *threshold. The finding for the relaxed set confirms the result reported by Han and Zhao [22]. Note, however, that *CpGcluster *strict set reaches the highest specificity but the lowest sensitivity. Interestingly, a recent study [29] also emphasizes that the *CpGcluster p-value *is a key attribute for distinguishing between constitutively methylated and unmethylated CGIs.

**Table 3:** Prediction of unmethylated regions (Bird's islands, N = 17,383).

Method	Number of predicted islands	Number of islands overlapping a Bird's island	Number of Bird's islands 'touched' by the prediction	SN	PPV
TJ	37,293	14,315	14,942	0.854	0.384

UCSC	27,639	13,858	14,256	0.816	0.501

CpGproD	76,886	14,250	15,346	0.875	0.185

*CpGcluster*:					

relaxed set*	198,702	29,235	15,497	0.939	0.147

strict set**	25,454	14,809	12,623	0.757	0.582

*p-value ≤ 1E-5; **p-value ≤ 1E-20

**Table 4:** Prediction of unmethylated regions (Weber's regions, N = 13,277).

Method	Number of predicted islands	Number of islands overlapping a Weber's region	Number of Weber's regions 'touched' by the prediction	SN	PPV
TJ	37,293	10,179	9,965	0.755	0.273

UCSC	27,639	9,788	9,552	0.724	0.354

CpGproD	76,886	10,320	10,257	0.774	0.134

*CpGcluster*:					

relaxed set*	198,702	18,967	10,372	0.867	0.095

strict set**	25,454	9,633	8,378	0.663	0.378

*p-value ≤ 1E-5; ** *p-value *≤ 1E-20

### CpG islands in the domains bound by polycomb repressive complex 2

Functional clusters of CpGs are not limited to promoter regions, they are also found in other genomic locations. An example are the hyperconserved CpG domains largely overlapping the domains bound by polycomb repressive complex 2 (PRC2) [30], located far from the promoter and playing an important role in transcriptional silencing during development. We determined the overlap of the CGIs predicted by different finders with the domains bound by PRC2. Table 5 shows that all the finders show high sensitivities and low PPVs in predicting these sites, being *CpGcluster *the algorithm obtaining the highest sensitivity (relaxed set).

**Table 5:** Overlap of different CGIs with 3,465 domains bound by the polycomb repressive complex 2 (PRC2).

Method	Number of predicted islands	Number of islands overlapping PRC2 domains	Number of PRC2 domains 'touched' by the prediction	SN	PPV
TJ	37,293	3,523	3,033	0.891	0.094

UCSC	27,639	3,179	2,790	0.825	0.115

CpGproD	76,886	3,321	3,159	0.916	0.043

*CpGcluster*:					

relaxed set*	198,702	9,097	3,097	0.961	0.046

strict set**	25,454	3,424	2,372	0.758	0.135

*p-value ≤ 1E-5; **p-value ≤ 1E-20

### Functional specificity vs. length of CpG islands

**Figure 6:**
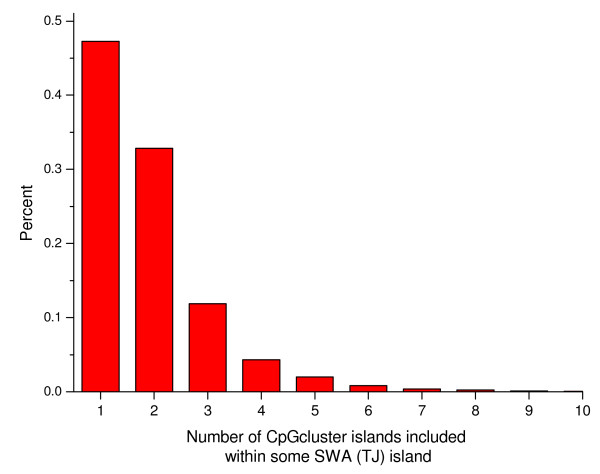
**Distribution of the number of *CpGcluster *islands included within SWA (TJ) islands**.

One of the most striking differences between SWA and the *CpGcluster *approach is the length of the predicted islands. SWA islands are on average much longer than *CpGcluster *islands (TJ = 1,094.9; UCSC = 764.5; CpGProD = 1,046.1; *CpGcluster *= 273.2 (relaxed set), or 727.5 (strict set)). Originally, CGIs were estimated to be on average 1 kb long [1]. Frequently, more than one *CpGcluster *island can be found within the promoter region and furthermore, several *CpGcluster *islands are often embedded within one single conventional, SWA island. For instance, around 53% of all TJ islands host more than one *CpGcluster *island (Figure 6).

Given these facts, it might be that either conventional SWA predictions erroneously merge smaller islands into longer ones, or that *CpGcluster *erroneously fragments longer islands into many smaller ones. Next, we use alternative TSSs and single CpG resolution methylation data to shed light on these questions.

#### Alternative promoters

Frequently, *CpGcluster *predicts more than one island within the promoter region. It has been shown [22] that 37.8% of all RefSeq genes have more than one *CpGcluster *island, while only 3.2% have more than one TJ island. Following the premise "one promoter one CpG island", this observation was interpreted as a disadvantage of *CpGcluster *[22]. However, in recent years, new insights into the regulation of gene expression became available, showing among other things a frequent use of alternative TSSs. The existence of alternative TSSs opens the possibility that more than one island per gene might exist. Therefore, the high percentage of genes with more than one *CpGcluster *island might instead indicate a more specific relation of *CpGcluster *islands to alternative promoters or TSSs. To check this possibility, we used the DBTSS database [31]. Out of 15,194 RefSeq genes annotated in the latest DBTSS release, 7,895 (52%) have at least one alternative TSS. With such scenario, one might expect up to 52% of all promoters having more than one island in its promoter (one for each TSS). Given these numbers, the reported 37.8% of genes with more than one *CpGcluster *island might look not so inadequate.

Conversely, this finding might indicate that the TJ algorithm artificially joins several functional islands into one single longer island. To further investigate this possibility, we estimated the number of islands simultaneously overlapping multiple TSSs annotated in the DBTSS database. Table 6 shows that the *CpGcluster *sets, both relaxed and strict, overlap a higher fraction of unique, and a lower fraction of multiple TSSs than the islands predicted by other programs, thus making *CpGcluster *predictions much more specific in overlapping individual TSSs.

**Table 6:** Co-localization of CpG islands and alternative promoters.

	Numbers of overlapping islands		
**Method**	**All the TSSs**	**Unique TSS**	**Multiple TSSs**

TJ	13,759	8,868 (64.45%)	4,891 (35.55%)

UCSC	11,826	8,143 (68.86%)	5,518 (31.14%)

CpGproD	15,319	9,801 (63.98%)	5,518 (36.02%)

*CpGcluster *islands:			

relaxed set*	15,095	12,034 (79.72%)	3,061 (20.28%)

strict set**	10,325	7,659(74.18%)	2,666 (25.82%)

*p-value ≤ 1E-5; ** *p-value *≤ 1E-20

**Figure 7:**
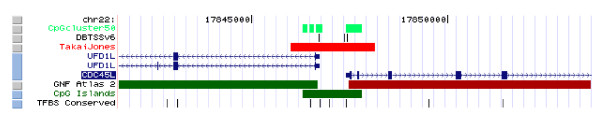
**A bidirectional promoter region in human chromosome 22 which is overlapped by one TJ or UCSC island but by several *CpGcluster *islands**. The two genes show very different expression profiles, and therefore it is very likely that the prediction of different islands for the different TSSs as done by *CpGcluster *is the better choice. The figure was obtained by using the UCSC Genome Browser [46].

Figure 7 shows a particular example of a bidirectional promoter region. The TSSs of the two genes, UFD1L and CDC45L, are overlapped by the same TJ or UCSC island, while *CpGcluster *predicts separate islands. This is interesting, as these two genes have very different expression breadths. Using the GeneAtlas2 expression data [32], we determined for UFD1L an expression breadth of 97.3% (expressed in 71 out of 73 healthy tissues), being therefore a housekeeping gene, while the CDC45L gene is expressed in just 15.1% (11 of 73) of all tissues. Given this differential gene expression pattern, a shared CpG island seems to be less specific than the scenario where each of the genes has its own island, as suggested by the prediction of *CpGcluster*.

In the human genome, there are a total of 166 bi-directional promoter pairs which share one long SWA CGI but two separated *CpGcluster *CGIs. The gene-pair shown in Figure 7 may be just an example of extreme differentiation in gene-expression: while the first member of the gene-pair is a housekeeping gene, the second one is a tissue-specific gene. However, one cannot reasonably expect that this may be the rule for all the bidirectional gene-pairs. In fact, after analyzing the expression profiles in a sample of 73 healthy tissues, only 16 (or 9.64%) gene-pairs show a completely divergent pattern of gene-expression (coexpression value ≤ 0.2, see Methods), while 13 (or 7.83%) exhibit complete coexpression (coexpression value = 1). The remaining gene-pairs show intermediate values of coexpression.

On the other hand, by using single base resolution methylation data [33], we also analyzed methylation differences between the CGIs overlapping bi-directional promoters. We found that 10 (or 11.24%) of these island-pairs in H1 stem cells, and 15 (or 16.85%) in the IMR90 fetal lung fibroblasts, show significant differences (Mann-Whitney non-parametric test) in their methylation average (p ≤ 0.05).

#### Heterogeneous methylation in long SWA islands

A functional CpG island should show a rather homogenous methylation profile among the different CpGs and over the different tissues. For example, the existence of more than one methylation domain within a predicted island might indicate an erroneous merging of two small islands into a single longer island.

**Figure 8:**
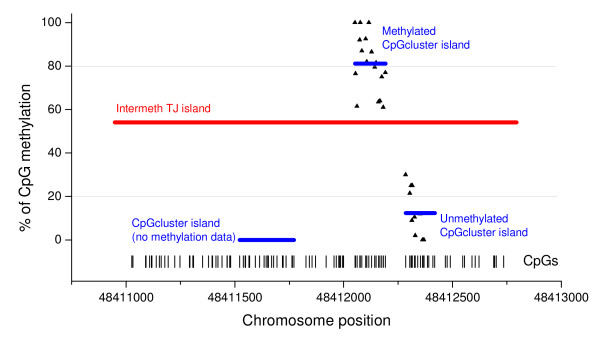
**A 317 bp long region of human chromosome 22 showing strong heterogeneity in methylation**. *CpGcluster *predicts separate islands for each methylation domain, while TJ and all the remaining tested sliding-window approaches predict only one longer island overlapping the different methylation domains.

Here, we used single base resolution methylation data from different sources (see Data and Methods) to decide whether *CpGcluster *predicts too many short islands or SWA predict too many long islands. In doing so, we detect all TJ islands which harbor at least two *CpGcluster *islands. Next, we calculate the mean methylation for each *CpGcluster *island and the maximal difference in methylation over the different tissues. If many TJ islands exist with high methylation differences inside, this might indicate an erroneously joining of different methylation domains into a single island. Figure 8 shows a particular example from human chromosome 22. The region for which HEP data were available is just 317 bp long, showing a very pronounced change of the methylation values in embryonic liver cells. All SWA programs predict a very long island in this region, including completely the interesting region where the un-methylation/methylation border occurs. Only *CpGcluster *predicts precisely one CGI for each of the methylation domains.

**Figure 9:**
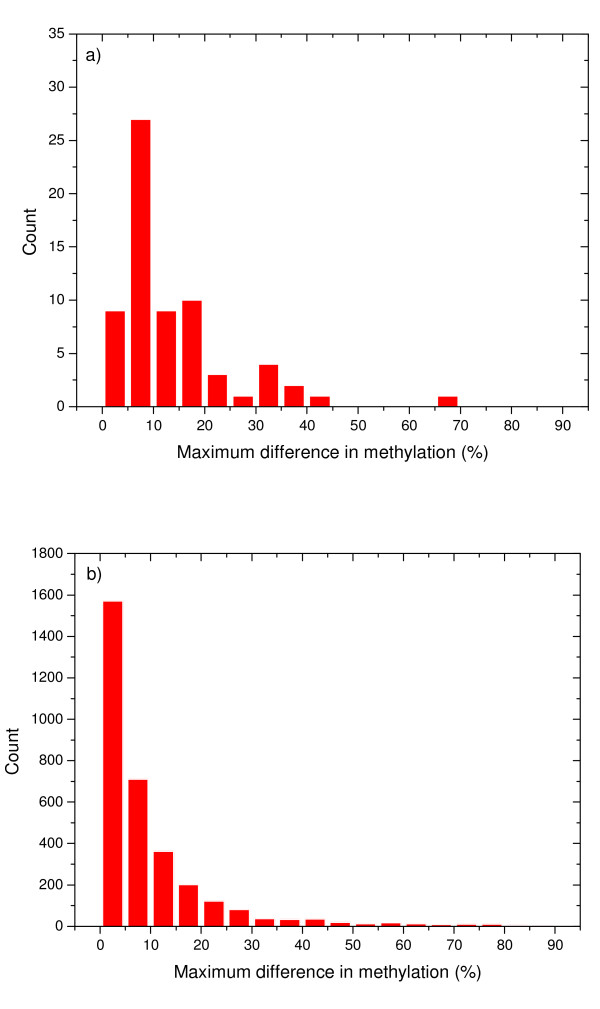
**Distribution of the maximal methylation differences between *CpGcluster *islands within SWA (TJ) islands**. a) HEP methylation data; b) Lister's methylation data [33].

Figure 9a shows the distribution of the maximum differences in the methylation of CpGs inside TJ islands for HEP data. It can be seen that very high differences occur, around 12% of all tested islands having higher differences than 30% in methylation. Methylation HEP data are available for only 5% of all tissues, and therefore the 12% of heterogeneous TJ islands merging several methylation domains might increase when data for more tissues becomes available. A similar conclusion can be reached when methylation data for two human methylomes [33] were used (Figure 9b). Note that the complex methylation structure within CpG islands has been reported before within a different context, but also showing that many long CpG islands contain more than one methylation domain [34].

### CpG-islets

CpG "islets", genomic regions not conventionally classified as CGIs because of their short length (<200 bp), but having a GC content and observed-to-expected CpG ratio characteristic of a CGI, have recently been identified in a 6.76-Mbp chromosomal region (10q25) containing a neocentromere [35]. Some of these islets remain unmethylated, corresponding to sites of active transcription and/or boundaries that separate major chromatin sub-domains. This suggests that, as conventional islands, the islets can also participate in the maintenance of a particular genomic pattern of methylated/unmethylated CpGs, thus contributing to the differential regulation of gene expression [3-5].

Given their tiny size, islets remain undetected by SWA, conventional CGI finders [2,21,36-40], as all these programs share a length threshold above 200, or even 500 bp. Such length thresholds make conventional finders useless for the detection of CpG islets, since a relaxation of the length threshold will lead to a strong increment of false positives. However, since *CpGcluster *[14] does not use any length threshold, it allows to identify short, but statistically significant CpG islets. A genome-wide search identifies a total of 88,137 CpG islets in the human genome with *p-value *≤ 10E-5. Table 7 shows that relatively high percentages of CpG islets overlap with different sets of promoters and evolutionarily conserved elements, thus suggesting a functional role for many of the predicted islets. Noteworthy, a high proportion of these overlapping islets are exclusively predicted by *CpGcluster*, but not by any of the remaining finders. This indicates that: 1) many of the small islands predicted by *CpGcluster *are not fragments of conventional islands, and 2) given the co-localization with functional regions, the islets might be indeed functional.

**Table 7:** Overlap of CpG islets (N = 88,137) with different sets of promoters and evolutionarily conserved elements.

Genome element	Number of overlapping CpG islets	Number of overlapping CpG islets exclusively predicted by CpG cluster
Promoters from RefSeq database	9,826 (11.15%)	1,218 (12.40%)

TSSs from DBTSS database	1,868 (2.12%)	398 (21.31%)

Promoter regions from DBTSS database	6,510 (7.39%)	4,869 (74.79%)

PhastCons	17,613 (19.98%)	8,219 (46.66%)

Using HEP data [41,42] and Lister et al. methylation levels of single cytosines [33], we also determined the number of unmethylated and differentially methylated CpG 'islets' (Table 8). A high proportion of the sampled CpG islets were unmethylated or differentially methylated, thus again suggesting a functional role for CpG islets. This is a very important point, as differential methylation of islands/islets may be involved in the regulation of gene expression. Again, the proportion of these CpG islets exclusively predicted by *CpGcluster *is very high.

**Table 8:** Number of unmethylated and differentially methylated CpG 'islets'.

Dataset	Methylation state*	Number of CpG islets	CpG 'islets' exclusively predicted by CpGcluster
HEP (12 tissues)**	Unmethylated	126	1

	Differentially methylated	26	8

Lister et al. 2009 (2 cell lines)***	Unmethylated	4,460	1,472
	
	Differentially methylated	373	295

*Unmethylated: average methylation ≤ 0.2; differentially methylated: average methylation <= 0.2 in at least one tissue & average methylation >= 0.8 in at least one other tissue.

**The methylation state of 246 CpG 'islets' from chromosomes 6, 20 and 22 was determined by using 3,168 individual CpG sites (HEP project). We only included CpGs which have been detected in at least 2 clones or in at least 6 different tissues.

***We used the sequence reads obtained by MethylC-Seq for two human cell lines [33], H1 human embryonic stem cells and IMR90 fetal lung fibroblasts, to get the average methylation level of single cytosines at both DNA strands for these two methylomes. All islands need more than 50% of its CpGs covered. Only cytosines covered by at least 10 reads were counted.

## Conclusions

We systematically compared conventional SWA for detecting CGIs to a clustering method, namely the *CpGcluster *algorithm. We showed than both approaches perform very similar when predicting long, unmethylated regions or polycomb sites. However, we found three scenarios where the *CpGcluster *algorithm seems to have advantages. First, the statistical significance assigned to each *CpGcluster *island seems to be a better quality parameter than the window size of conventional finders, as it reduces more efficiently false positive predictions. Second, we have shown that *CpGcluster *islands co-localize in a more specific way to alternative TSSs and methylation domains. Third, we have shown that many of the small islands predicted by *CpGcluster *might be functional, given the overlap with conserved elements or promoter regions. Moreover, 30% of the differentially methylated islets are exclusively predicted by *CpGcluster*, which suggests this method as the option of choice for the experimental verification of islet functionality.

## Methods

### Sequence Data

We used human genome assembly NCBI 36.1 (hg18), downloaded from the UCSC genome browser http://hgdownload.cse.ucsc.edu/downloads.html#human.

### Promoter data

To quantify the co-localization of the predictions with promoter regions and principal transcription start sites we used the RefSeq gene annotation [43]. We furthermore used the DBTSS database version 6.0 [44], as it annotates also alternative transcription start sites, as well as start sites which cannot be assigned to a known RefSeq transcript. From both, the RefSeq and DBTSS annotation, we extracted the coordinates of two regions; the transcription start site (TSS) and the promoter regions, defined as TSS-1500 bp to TSS+500 bp.

### Genomic elements

We determined the overlap of CGIs with conserved elements (PhastCons) and spurious *Alu *elements. The evolutionarily conserved elements [23] and the *RepeatMasker *[45] annotation of repeated elements where downloaded from the UCSC table browser [46]. In general, we consider two measures to quantify the overlap between CGIs and genomic elements. First, we define the mean coverage of a CGI prediction as the mean value of all coverage fractions. The coverage fraction can be calculated as the number of bases of an island corresponding to a given genomic element divided by the island length. Furthermore, we calculate the overlap fraction as the number of islands which overlap in at least one base with a given genomic element divided by the total number of predicted islands.

### Island predictions

For SWA CGI finders, a CpG island was at least 200 bp long, which excluded the detection of any shorter tracts. To detect CpG-rich regions, disregarding its length, we used a recently published CpG island finder algorithm (*CpGcluster*, [14]) which does not rely on any length threshold but directly predicts statistically significant CpG clusters. Briefly, the *CpGcluster *algorithm can be divided into two steps. First, based on a distance threshold, the individual CpGs which are below this threshold are clustered along the DNA sequence. Second, by means of the negative binomial distribution a *p-value *is assigned to each CpG cluster, which allows the prediction of highly significant clusters such as CpG islands.

We considered five computational predictions of CpG islands. For the *CpGcluster *algorithm [14], we generated two prediction sets by setting the assigned *p-value *to two different thresholds. We generated a relaxed set with *p-value *<= 1E-5 and a strict set by setting the threshold to 1E-20. We implemented the TJ algorithm, as explained in [21], by setting the thresholds to: length ≥ 500 bp, GC content ≥ 55%, Obs_CpG_/Exp_CpG _≥ 0.65 and minCpG >= 0.6*L_island_/16 (to avoid "mathematical" islands). We generated the CpGproD prediction [38] running the program http://pbil.univ-lyon1.fr/software/cpgprod.html with default parameters. Finally, we downloaded the UCSC CpG island predictions from the UCSC table browser [46].

### Gene coexpression analysis

We used the GeneAtlas2 expression data [32] to determine the co-expression of gene pairs sharing a bi-directional promoter. The "coexpression value" for a couple of genes is the ratio of the number of tissues in which both genes are simultaneously expressed (signal levels > 200) or simultaneously not expressed (signal levels <= 200), and the number of healthy tissues with expression data.

### Methylation data

Since the lack of methylation of a CpG island is a very good indicator of function [25], we used several different sources of experimental methylation data. Weber *et al*. [26] detected methylation states in two different tissues, fibroblast and sperm. We extracted 13,277 non-overlapping regions which are unmethylated in at least one of the two tissues (scaled 5mC log2 ratio < 0.3). Next, we used 17,383 CpG island recently detected in blood cells by means of a new technique [28].

Finally, we assigned methylation states (unmethylated, methylated and differentially methylated) to our *CpGcluster *predictions by means of the data from the HEP-human epigenome project [42]. The data comprises about 1.9 million CpG methylation values, obtained from the analysis of 2,524 amplicons across chromosomes 6, 20 and 22 in 43 samples (derived from 12 different tissues). We first calculated the mean methylation of each CpG dinucleotide over the different clones, then deleting all CpGs which have been detected in less than 2 clones or in less than 6 different tissues. Subsequently, the individual CpGs were labeled as methylated (mean methylation >= 80), intermediate methylated (80-20) and unmethylated (under 20) for each of the different tissues. Next, we define the methylation states of the CpGs over the different tissues in the following way: i) methylated CpG: methylated in more than 50% of tissues and never unmethylated, ii) unmethylated CpG: unmethylated in more than 50% of tissues and never methylated, iii) differentially methylated CpG: both, methylated and unmethylated in different tissues, the number of intermediate methylation states being smaller than 50%. Finally, we assign a methylation label to the CpG islands which have methylation data for more than 50% of its CpGs: i) methylated: more than 50% of the CpGs are methylated and no unmethylated CpG exist, ii) unmethylated: more than 50% of the CpGs are unmethylated and no methylated CpG exist, iii) differentially methylated: more than 50% of all CpGs need to be differentially methylated.

We also used the sequence reads obtained by MethylC-Seq for two human cell lines [33], H1 human embryonic stem cells and IMR90 fetal lung fibroblasts, to get the average methylation level of single cytosines at both DNA strands for these two methylomes. All islands need more than 50% of its CpGs covered. Only cytosines covered by at least 10 reads were counted.

### Assessing prediction quality

When comparing the prediction of CpG islands to a gold standard (e.g. experimentally verified islands), we define:

• True Positives (TP): An island overlapping in at least 1 bp with the gold standard

• False Positives (FP): An island not overlapping with the gold standard

• False Negative (FN): An island in the gold standard that has not been predicted.

By means of these values, we then calculate the sensitivity and the Positive Predictive Value (also known as specificity in the gene prediction field [27]):

Note that we consider all islands not overlapping with the gold standard as false positive predictions. However, no complete gold standard exists, and therefore an unknown number of these islands will be actually true positive predictions. This assumption does not affect the sensitivity, as FP does not occur in the equation, but it affects the PPV. Consequently, and since the PPV can only increase when some FPs turn out to be TPs, the value used in this work is the lower boundary PPV of the prediction, e.g. the worst case scenario when all islands which do not overlap with the gold standard are indeed false positives.

## List of abbreviations

CGI: CpG island; CpG O/E ratio: Ratio between observed and expected CpG frequencies; CpG: dinucleotide CG; G + C content, %G + C: Molecular fraction of guanine and cytosine; PhastCons: Phylogenetic Conserved Elements; Sn: The sensitivity of the prediction; PPV: Positive Predictive Value of the prediction; SWA: Sliding-window approaches; TJ: Takai/Jones program or island; TSS: Transcription Start Site

## Competing interests

The authors declare that they have no competing interests.

## Authors' contributions

MH designed and performed the experiments and wrote the manuscript (with JLO), GB performed the search for alternative promoters and differential methylation, PC and PLE carried out the theoretical analysis of CpG clustering and help with the interpretation of statistical results, CP determined the overlap of the CGIs predicted by different finders with the domains bound by polycomb repressive complex, and JLO designed the experiments and wrote the manuscript (with MH). All the authors critically read and approved the final version.
